# An update on psoriasis and metabolic syndrome: A meta-analysis of observational studies

**DOI:** 10.1371/journal.pone.0181039

**Published:** 2017-07-18

**Authors:** Sanminder Singh, Paulina Young, April W. Armstrong

**Affiliations:** 1 University of California, Davis School of Medicine, Sacramento, California, United States of America; 2 Department of Dermatology, Keck School of Medicine, University of Southern California, Los Angeles, California, United States of America; Weill Cornell Medical College in Qatar, QATAR

## Abstract

The relationship between psoriasis and metabolic syndrome is not well understood. Though multiple epidemiologic studies have suggested a link between psoriasis and metabolic syndrome, there is a lack of a comprehensive meta-analysis synthesizing the results of all available observational studies to date. In this meta-analysis, we examined global data on the relationship between psoriasis and odds of metabolic syndrome by searching for studies published between 1946–2016. Specifically, we analyzed the results from 35 observational studies from 20 countries with 1,450,188 total participants, of which 46,714 were psoriasis patients. The pooled odds ratio based on random effects analysis was 2.14 (95% CI 1.84–2.48). Publication bias was present, as evidenced by an Egger test and graphical visualization through a funnel plot (p = 0.001). Based on this comprehensive meta-analysis, psoriasis patients have higher odds of having metabolic syndrome when compared with the general population.

## Introduction

Psoriasis is a chronic, inflammatory skin condition that affects between 2–4% of the general population, with recent estimates suggesting over 125 million patients worldwide [1, 2]. It is associated with a number of comorbidities as well as a high socioeconomic burden [3, 4]. Psoriasis patients also experience a decreased quality of life as a result of this disease [5]. The pathogenesis of psoriasis is currently under active investigation, with studies aiming to identify genetic susceptibility loci for psoriasis in order to detect novel targets for systemic therapy [6–9]. While the exact pathogenesis of psoriasis is not fully understood, basic and translational investigations have led to a renewed understanding of Th-17 and Th-1 pathways involved in the development of psoriasis [10]. Notably, the Th-1 pathway involving dysregulation and activation of Th-1 inflammatory cells is thought to contribute to obesity and insulin resistance, which can increase the risk for cardiovascular disease [11–13].

Population studies have found that patients with psoriasis have increased prevalence of cardiovascular risk factors and elevated risk for developing adverse cardiovascular outcomes [14, 15]. Among these studies, investigators have sought to identify the relationship between psoriasis and metabolic syndrome.

Metabolic syndrome is composed of an assortment of metabolic abnormalities that augment the risk of developing cardiovascular disease. The prevalence of metabolic syndrome is 35% in the United States [16] and has been associated with a significant economic burden [17, 18]. In addition to economic costs, metabolic syndrome is associated with a greater risk of cardiovascular mortality [15, 19]. Specifically, according to NCEP ATP III criteria, metabolic syndrome is diagnosed when a person has at least three of these five conditions: 1) waist circumference 102 cm (40 inches) or greater in men or 88 cm (35 inches) or greater in women; if Asian American, 90 cm (35 inches) or greater in men or 80 cm (32 inches) or greater in women, 2) triglycerides 150 mg/dL or higher (or receiving drug therapy for hypertriglyceridemia), 3) high-density lipoprotein- cholesterol complex (HDL-C) less than 40 mg/dL in men or less than 50 mg/dL in women (or receiving drug therapy for reduced HDL-C), 4) blood pressure 130/85 mm Hg or higher (or receiving drug therapy for hypertension), and 5) fasting glucose 100 mg/dL or greater (or receiving drug therapy for hyperglycemia) [20, 21].

Psoriasis and metabolic syndrome may develop interdependently due to a shared immunopathogenesis involving chronic low-level inflammation mediated by pro-inflammatory cytokines such as IFN-gamma, IL-17, IL-23, and TNF-alpha [14, 22–24]. Additionally, some studies have implicated insulin-like growth factor 1 (IGF-1) as a shared mediator in the keratinocyte proliferation seen in psoriasis and the development of diabetes and hyperlipidemia [25, 26].

Several observational studies have suggested an epidemiological link between psoriasis and metabolic syndrome. There is a lack of comprehensive synthesis of observational data that explores the relationship between these two diseases. To examine whether patients with psoriasis are more likely to have metabolic syndrome, we analyzed data from all available observational studies examining the relationship between psoriasis and metabolic syndrome from 1946–2016.

## Materials and methods

To examine the association between metabolic syndrome and psoriasis, we followed PRISMA guidelines to perform a meta-analysis of observational studies identified through our two prior systematic reviews [27, 28] combined with additional studies from a new literature search. The PRISMA checklist is attached (S1 File). For the prior systematic reviews, we used the search terms “Psoriasis” [MeSH] and “Metabolic Syndrome X” [MeSH] to search literature from January 1, 1946 to June 30, 2016. To search for any additional studies published after June 30, 2016, we repeated the search using the same search terms and applied a time restriction for studies published from July 1, 2016 to Jan 1, 2017.

**Fig 1:**
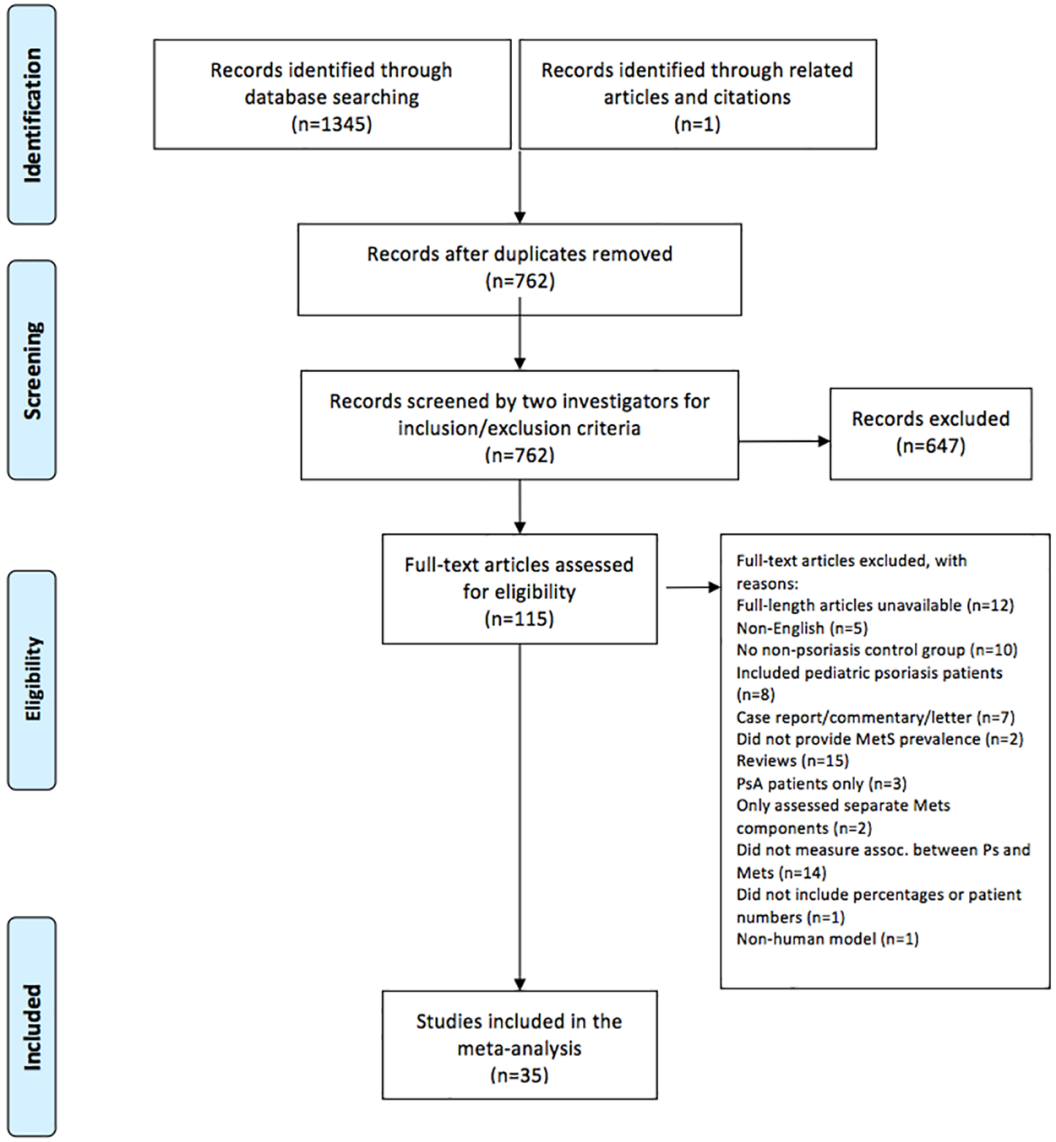
Selection process for studies included in the meta-analysis.

From this updated search of the Medline, Embase, Cochrane Central Register, and Scopus databases, we yielded 1346 references. This was inclusive of 41 references identified through the new search. We applied the following inclusion/exclusion criteria to the 1346 references: human subjects, English language, and observational study design (case-control, cohort, cross-sectional, or nested case-control). Additionally, the studies must have provided prevalence or incidence data on metabolic syndrome in conjunction with psoriasis. The studies must have compared prevalence of metabolic syndrome in healthy or non-psoriasis controls. The diagnosis of psoriasis and metabolic syndrome must be made by a physician and documented in the medical record. Studies evaluating pediatric patients were excluded, as were review articles, commentaries, case reports, case series, and letters to the editor (Fig 1).

Once duplicates were removed and initial screening performed by two authors, we performed a full-length review of 115 references and excluded 80 for the following reasons: full-length articles unavailable (n = 12), non-English (n = 5), no non-psoriasis control group (n = 10), included pediatric psoriasis patients (n = 8), case report/commentary/letter (n = 7), did not provide metabolic syndrome prevalence (n = 2), reviews (n = 15), PsA patients only (n = 3), only assessed separate metabolic syndrome components (n = 2), did not measure association between psoriasis and metabolic syndrome (n = 14), did not include percentages or patient numbers (n = 1), and focused on non-human model (n = 1). While there were some case series/reports relevant to this topic, we focused our systematic review and meta-analysis on observational studies. We selected six new references to be systematically reviewed in this paper. For the meta-analysis, we drew upon 29 studies from our previous systematic reviews and added six additional studies from an updated search, for a total of 35 studies to be analyzed.

We extracted prevalence of metabolic syndrome in psoriasis patients versus controls as well as the reported effect size where available, such as odds ratios. Of the 35 studies included in the meta-analysis, 16 reported unadjusted odds ratios, 10 reported adjusted odds ratios, seven studies provided prevalence rates as percent values without calculated odds ratios, and two reported number of patients with metabolic syndrome without percent values or odds ratios. For studies without a published odds ratio value, we calculated unadjusted odds ratios using STATA 14.1 [29]. In studies where investigators provided multiple odds ratios for subsets of psoriasis patients without an overall odds ratio, we used all reported odds ratios for the meta-analysis [30, 31].

Using odds ratio data obtained from the published results, we estimated the pooled odds ratio for the presence of metabolic syndrome in psoriasis patients. To account for any study heterogeneity, we utilized the random-effects model of DerSimonian and Laird [32].

We performed an Egger’s regression test to assess for publication bias. Publication bias may arise when studies with statistically significant results are more likely to be published and cited, and are preferentially published in English language journals [33]. Studies that do not suggest a relationship between psoriasis and metabolic syndrome may be less likely to get published, thus this Egger’s regression test enables us to assess the degree of publication bias in this field of investigation. Specifically, we graphically represented the estimate of effect from each study in the meta-analysis against a measure of its precision, producing a funnel plot, and a bias coefficient was calculated to confirm the findings of the funnel plot. We also examined between-study heterogeneity using the I^2^ statistic.

All analyses were performed using STATA Version 14.1 (STATA Corp LP, College Station, TX).

## Results

This meta-analysis analyzed data from 35 studies with a total of 1,450,188 participants, among which 46,714 were patients with psoriasis. We searched over 70 years of literature, spanning from January 1, 1946 to Jan 1, 2017, using the Medline, Embase, Cochrane Central register, and Scopus databases [27, 28]. Out of the 35 articles, ten provided adjusted odds ratios after multivariate adjustment for factors such as age, sex, smoking, alcohol consumption, physical activity, and education [31, 34–42]. Among the remaining articles, 16 provided unadjusted odds ratios [30, 43–58], seven provided percent values for metabolic syndrome prevalence [59–65], and two provided specific numbers of patients affected without percent values or odds ratios. For the nine articles that did not provide any odds ratios, we calculated unadjusted odds that were then included in the meta-analysis (Table 1).

**Table 1:** Study characteristics and outcomes for psoriasis and metabolic syndrome.

Study	Study Setting	Method of assessing metabolic syndrome	No. of psoriasis patients, n	No. of controls, n	No. of patients with Metabolic Syndrome in Psoriasis, n (%)	No. of patients with Metabolic Syndrome in controls (%)	Odds of having metabolic syndrome in psoriasis patients (95% CI)
Sommer et al. (2006)[54]	Germany; inpatient (hospital charts)	Manual chart review	581	1044	25 (4.3)	11 (1.1)	OR 4.22 (2.06–8.65)
Gisondi et al. (2007)[49]	Italy; outpatient (outpatient clinics)	Clinical assessment; NCEP ATP III criteria	338	334	102 (30.1)	69 (20.6)	OR 1.65 (1.16–2.35)
Chen et al. (2008)[47]	Taiwan; outpatient (dermatology clinics)	Clinical assessment	77	81	10 (14.1)	13 (16.3)	OR 0.84 (0.31–2.26)
Chen et al. (2009)[46]	Taiwan; outpatient (dermatology clinics)	Clinical assessment	40	37	9 (22.5)	4 (10.8)	OR 2.40 (0.67–8.58)
Nisa and Qazi (2010)[51]	India; outpatient (dermatology clinics)	Clinical assessment; NCEP ATP III criteria	150	150	42 (28.0)	9 (6.0)	OR 6.09 (NR)
Takahashi et al. (2010)[55]	Japan; outpatient (dermatology clinics)	Manual chart review	151	154	38 (25.2)	25 (16.2)	OR 1.74 (0.99–3.05)
Al-Mutairi et al. (2010)[30]	Kuwait; outpatient (medical records)	Manual chart review	1835	1835	Mild-moderate psoriasis: 265 (16.0) Severe psoriasis: 34 (26.4)	124 (6.8)	Mild psoriasis: OR 2.62 (2.09–3.28) Severe psoriasis: OR 4.93 (3.21–7.60)
Augustin et al. (2010)[44]	Germany; outpatient (health insurance database)	ICD-10 codes	1,310,090	33,981	61 (0.2)	786 (0.1)	OR 2.86 (2.21–3.71)
Bongiorno et al. (2010)[45]	Italy; outpatient (dermatology clinics)	Clinical assessment; NCEP ATP III criteria	400	348	103 (25.8)	32 (9.2)	OR 3.4 (2.23–5.24)
Balci et al. (2010)[60]	Turkey; outpatient (dermatology clinics)	Clinical assessment; NCEP ATP III criteria	46	46	8 (17)	5 (11)	OR 1.73 (0.52–5.75)*
Mebazaa et al. (2011)[39]	Tunisia; outpatient (dermatology clinics)	Clinical assessment; NCEP ATP III criteria	164	216	67 (40.9)	67 (31.0)	OR 1.39 (0.88–2.18) AOR 1.73 (1.06–2.82)
Love et al. (2011)[38]	United States; outpatient (NHANES)	Clinical assessment; NCEP ATP III criteria	71	2385	28 (39.9)	560 (23.5)	OR 2.16 (1.16–4.03) AOR 1.96 (1.02–3.77)
Zindanci et al. (2012)[56]	Turkey; NR (dermatology department)	Clinical assessment; IDF criteria	115	140	61 (52)	59 (39)	OR 2.94 (1.40–6.19) PASI <10: 51% PASI >10: 56%
Arias-Santiago et al. (2012)[43]	Spain; outpatient (dermatology department)	Clinical assessment; NCEP ATP III criteria	72	61	29 (40)	8 (13)	OR 4.46 (1.85–10.72)
Madanagobalane et al. (2012)[62]	India; outpatient (dermatology clinics)	Clinical assessment; SAM-NCEP ATP III criteria	118	120	52 (44)	36 (30)	OR 1.84 (1.08–3.14)* PASI 0–7: 46% PASI 8–12: 30% PASI >12: 50%
Langan et al. (2012)[37]	United Kingdom; outpatient (THIN database)	Read codes (THIN database)	4065	40,650	1389 (34.2)	10,515 (25.9)	OR 1.50 (1.40–1.61) Overall AOR 1.41 (1.31–1.51) Mild psoriasis: AOR 1.22 (1.11–1.35) Moderate psoriasis: AOR 1.56 (1.38–1.76) Severe psoriasis: AOR 1.98 (1.62–2.43)
Vaya et al. (2013)[41]	Spain; outpatient (dermatology & rheumatology departments, transfusion center)	Clinical assessment; NCEP ATP III criteria	91	101	28 (31)	18 (18)	OR 2.05 (1.04–4.05) AOR 0.36 (0.10–1.25)
Damevska et al. (2013)[48]	Macedonia; inpatient & outpatient (dermatology clinics)	Clinical assessment; NCEP ATP III criteria	122	122	30 (25)	28 (23)	OR 1.09 (0.61–1.97) BSA <10%: 25%
Karoli et al. (2013)[61]	India; NR (dermatology department)	Clinical assessment; NCEP ATP III criteria	96	100	39 (40)	22 (22)	OR 2.43 (1.30–4.54)* PASI >12: 77%
Tasliyurt et al. (2014)[63]	Turkey; outpatient (dermatology clinics & internal medicine department)	Clinical assessment; NCEP ATP III criteria	37	28	14 (35)	4 (14)	OR 3.65 (1.05–12.74)*
Akcali et al. (2014)[59]	Turkey; outpatient (dermatology clinics)	Clinical assessment; NCEP ATP III criteria	50	40	25 (50)	10 (25)	OR 3.00 (1.21–7.42)*
Kokpol et al. (2014)[36]	Thailand; outpatient (dermatology clinics & NHES database)	Clinical assessment; IDF criteria	199	199	98 (49)	61 (31)	OR 2.19 (1.46–3.31) AOR 2.25 (1.35–3.75) PASI < 3: 45% PASI 3–9: 60% PASI >10: 29%
Parodi et al. (2014)[40]	Italy; outpatient (dermatology clinics)	Clinical assessment; NCEP ATP III criteria	390	344	102 (27)	52 (15)	AOR 1.96 (1.22–3.14) PASI <10: 20% PASI 10–19.9: 24% PASI >20: 34%
Irimie et al. (2015)[50]	Romania; inpatient & outpatient (dermatology department)	Clinical assessment; IDF criteria	142	167	19 (13)	18 (11)	OR 1.3 (0.64–2.54)
Danielsen et al. (2015)[34]	Norway; outpatient (Tromso study database)	Clinical assessment; adaptation of IDF, NHLBI, and other criteria	1137	9384	375 (33)	2346 (25)	OR 1.43 (1.25–1.63) AOR 1.35 (1.17–1.56)
Miller et al. (2015)[31]	Denmark; outpatient (dermatology department & GESUS database)	Self-report and clinical assessment; NCEP ATP III criteria	Hospital: 36 Population: 860	14,016	Hospital: 23 (66) Population: 226 (34)	3032 (27)	Hosp pso: AOR 5.14 (2.47–10.69) Pop pso: AOR 1.29 (1.09–1.53)
Itani et al. (2016)[35]	Lebanon; outpatient (dermatology clinics)	Clinical assessment; NCEP ATP III criteria	150	150	53 (35)	27 (18)	OR 2.49 (1.46–4.25) AOR 2.30 (1.34–3.95) PASI <10: 30% PASI >10: 49%
Praveenkumar et al. (2016)[52]	India; outpatient (dermatology department)	Clinical assessment; SAM-NCEP ATP III criteria	30	30	18 (60)	12 (40)	OR 2.25 (0.801–6.321)
Sharma et al. (2016)[53]	India; NR (dermatology department)	Clinical assessment; SAM-NCEP ATP III criteria	100	100	38 (38)	12 (12)	OR 4.49 (2.17–9.29) PASI >10: 35%
Barrea et al. (2016)[64]	Italy; outpatient (dermatology department)	Clinical assessment; NCEP ATP III criteria	180	180	117	57	OR 4.01 (2.59–6.22)*
Brito-Luna et al. (2016)[66]	Mexico; NR	Clinical assessment; NCEP ATP III criteria	55	30	31	16	OR 1.13 (0.46–2.76)*
Djurovic et al. (2016)[42]	Montenegro; outpatient (dermatology clinic)	Clinical assessment; NCEP ATP III criteria	101	126	49	26	AOR 2.99 (1.59–5.62)
Kothiwala et al. (2016)[58]	India; inpatient	Clinical assessment; NCEP ATP III criteria	140	140	55	24	OR 3.12 (1.79–5.44)
Lai et al. (2016)[57] ^†^[67]	United States; outpatient (NHANES)	Joint Scientific Statement Criteria (2009)	520	19065	108	2552	OR 1.69 (1.37–2.10)*
Vandervoort et al. (2016)[65]	Netherlands; outpatient (NR)	Clinical assessment; NCEP ATP III criteria	74	1461	39	679	OR 1.28 (0.80–2.04)*

*OR*, *odds ratio; AOR*, adjusted odds ratio; *BSA*, body surface area; *IDF*, International Diabetes Federation; *NCEP-ATP III*, National Cholesterol Education Program Adult Treatment Panel III; *NHLBI*, National Heart, Lung, and Blood Institute; *NR*, not recorded; *PASI*, Psoriasis Area Severity Index; *SAM-NCEP-ATP III*, South Asian Modified National Cholesterol Education Program Adult Treatment Panel III; THIN, The Health Improvement Network; IDF, International Diabetes Foundation; NHANES, National Health and Nutrition Examination Survey; NHES, Thailand National Health Examination Survey; GESUS, Danish General Suburban Population Study.

* In studies that did not report odds ratios, unadjusted odds ratios were calculated using published results.

^†^ A subset of this population was reported in a different study

**Fig 2:**
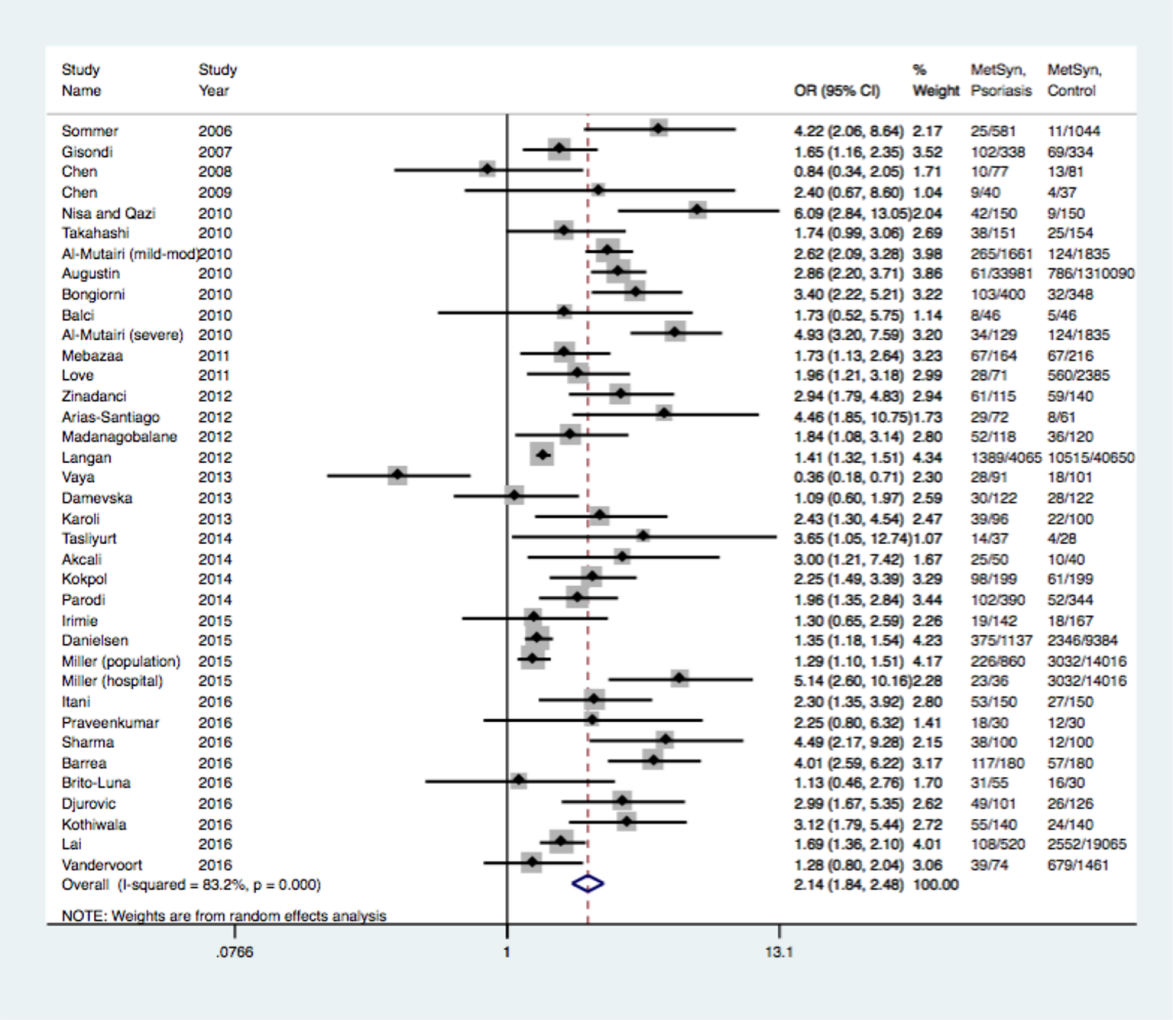
Prevalence of metabolic syndrome in psoriasis patients. This is a forest plot examining observational studies. The diamond represents the exact estimate from the study. The width of the line extending from each diamond represents the 95% confidence interval (CI). OR, odds ratio; MetSyn, Metablic syndrome.

All of the studies included in this meta-analysis reported the prevalence of metabolic syndrome in psoriasis patients versus non-psoriasis controls. With the inclusion of seven new studies published from July 1, 2016- January 1, 2017, the unadjusted odds ratios for metabolic syndrome in psoriasis patients ranged from 0.84–6.09, and the adjusted odds ratios ranged from 1.22–5.14. When stratified by disease severity, the prevalence of metabolic syndrome demonstrated a dose-dependent relationship with the degree of psoriasis severity [30, 37, 40, 56, 61, 62, 68]. For example, the adjusted odds ratio for metabolic syndrome in severe psoriasis was 1.98, while in mild psoriasis it was 1.22 [37]. For the meta-analysis, we utilized random effects analysis to calculate the pooled odds ratio of 2.14 (95% CI 1.84–2.48), as shown in Fig 2.

**Fig 3:**
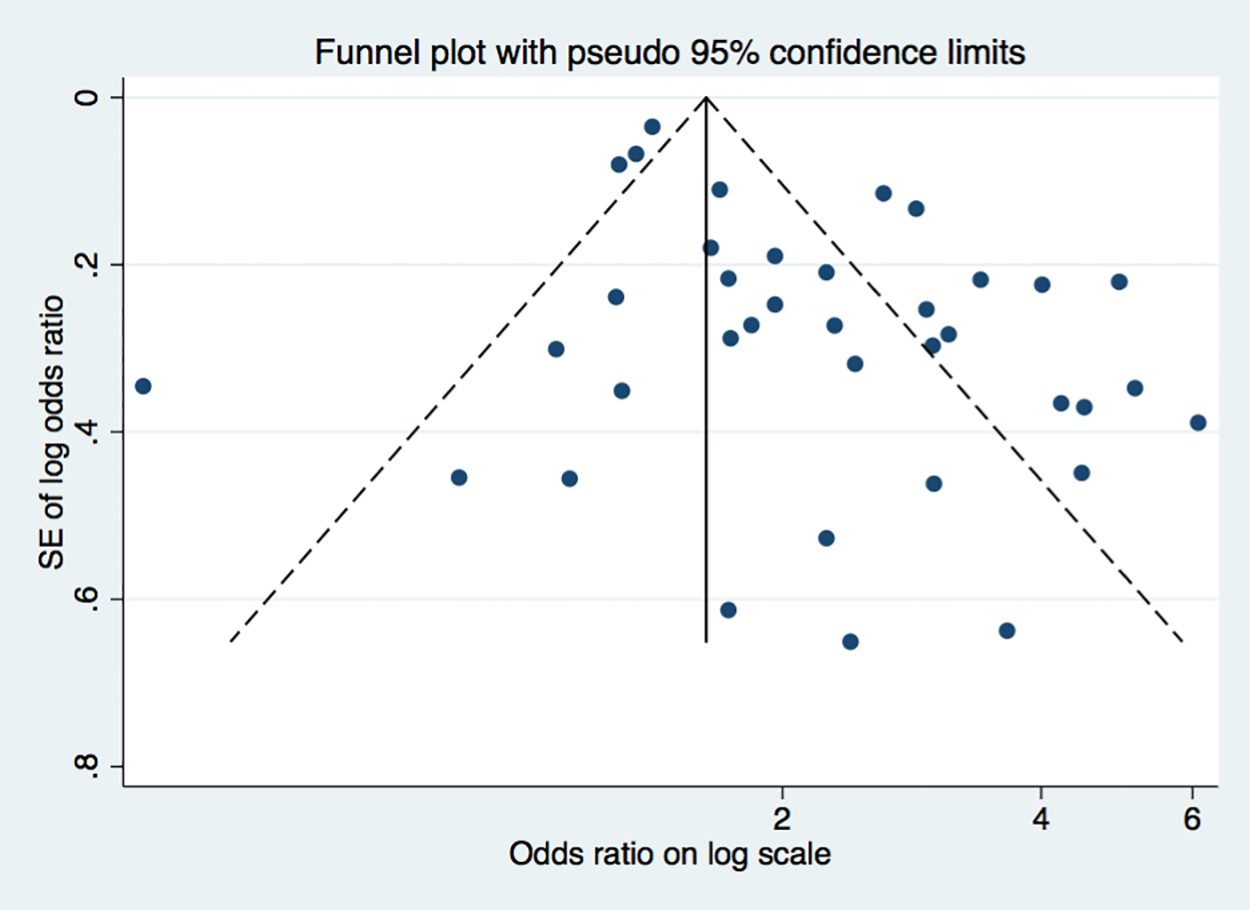
Funnel plot of included studies.

To assess publication bias, we examined a funnel plot and performed an Egger test to determine a bias coefficient (Fig 3). The funnel plot revealed asymmetry, suggesting publication bias, and the Egger test confirmed bias (P = 0.001). I^2^ value of 83.2% (p = 0.000) suggests presence of between-study heterogeneity.

## Discussion

This is a meta-analysis based on primary studies published between 1946–2017 that were identified through a comprehensive systematic review. The meta-analysis examines the relationship between psoriasis and metabolic syndrome, incorporating 35 articles from 20 countries with a total of 1,450,188 participants, including 46,714 psoriasis patients. To our knowledge, this is the most comprehensive meta-analysis on psoriasis and metabolic syndrome to date. Our pooled OR of 2.14 indicates a higher prevalence of metabolic syndrome in psoriasis patients in comparison to the general population. With the added impact of new and previously unexamined primary studies, our study maintains and strengthens the positive correlation between psoriasis and metabolic syndrome found in our 2013 meta-analysis, which had demonstrated a pooled odds ratio of 2.26 from 12 studies [27, 28].

Metabolic syndrome encompasses a group of cardiovascular risk factors that overlap with psoriasis in both pathogenesis and outcome. These include abdominal obesity, hypertension, dyslipidemia, and insulin resistance. Additionally, metabolic syndrome, along with several of its components when considered individually, increase in prevalence along with psoriasis disease severity [69].

Shared underlying mechanisms between psoriasis and metabolic syndrome have been proposed. One such theory centers on how the chronic inflammation in psoriasis also contributes to insulin resistance and endothelial cell dysfunction in atherosclerosis. This inflammatory cascade ultimately culminates in adverse cardiovascular events such as myocardial infarction or stroke [70].

Specifically, psoriasis and metabolic syndrome display similar inflammatory profiles with Th1 and Th17 T-cells as well as overexpression of cytokines such as IL-6 and TNF-alpha [69]. For example, TNF-alpha, which is overexpressed in patients with psoriasis, is also elevated with abdominal obesity, a component of metabolic syndrome. It induces the production of adhesion molecules by endothelial cells, promoting monocyte binding in the early phases of atherosclerosis [71]. If left uncontrolled, the immunologic mediators common to both disease processes may lead to cardiovascular impairment or death.

The results of the meta-analysis need to be interpreted in the context of the primary articles. One factor to consider in examining any meta-analysis is sources of heterogeneity. Heterogeneity can increase generalizability of the study findings, but it can also impact interpretation of the association between exposure and outcome. Aside from differences in study location, setting, and design, some studies reported unadjusted odds ratios while others reported adjusted odds ratios. Furthermore, the assessment of metabolic syndrome varied as well, with some studies using NCEP-ATP III criteria, while others using the South Asian Modified version of the NCEP-ATP III criteria or the International Diabetes Foundation (IDF) definition [72]. Therefore, we used random effects analysis to account for these sources of heterogeneity, and the odds of metabolic syndrome in psoriasis patients remained more than twice that of the general population.

As with most meta-analyses, in this meta-analysis, we detected publication bias through use of the Egger test. Therefore, the results of this study may reflect a slight overestimate of the frequency of metabolic syndrome in psoriasis patients. Nevertheless, the outcomes of a vast majority of the 35 studies evaluated suggest a positive and strong correlation between psoriasis and metabolic syndrome.

Through a thorough evaluation of existing literature, we found significantly increased odds of metabolic syndrome in the psoriasis population compared to non-psoriasis controls. We also found there to be a dose-dependent relationship between psoriasis disease severity and metabolic syndrome prevalence. Psoriasis is a systemic disease with significant morbidity and mortality. This study emphasizes the critical need for providers to screen psoriasis patients for cardiometabolic diseases and provide structured management. This may require dermatologists to work in concert with primary care providers and other specialists to coordinate care for psoriasis and its comorbidities. Finally, the exact pathologic mechanisms shared by these two disease processes, as well as the directionality of the relationship, are not well understand and need to be elucidated through further translational work. Future translational discoveries may guide the development of new treatments or the refined application of existing therapies for both psoriasis and metabolic syndrome.

## Supporting information

S1 File PRISMA checklist. (PDF)
